# Prior activity of olfactory receptor neurons is required for proper sensory processing and behavior in *Drosophila* larvae

**DOI:** 10.1038/s41598-018-26825-3

**Published:** 2018-06-05

**Authors:** Nao Utashiro, Claire R. Williams, Jay Z. Parrish, Kazuo Emoto

**Affiliations:** 1 Department of Biological Sciences, School of Science, The University of Tokyo, 7-3-1 Hongo, Bunkyo-ku, Tokyo, 113-0033 Japan; 2 Department of Biology, University of Washington, 24 Kincaid Hall, Box 351800, Seattle, WA 98195 USA; 3 Molecular and Cellular Biology Program, University of Washington, Seattle, WA 98195 USA; 4 International Research Center for Neurointelligence (WPI-IRCN), The University of Tokyo, 7-3-1 Hongo, Bunkyo-ku, Tokyo, 113-0033 Japan

## Abstract

Animal responses to their environment rely on activation of sensory neurons by external stimuli. In many sensory systems, however, neurons display basal activity prior to the external stimuli. This prior activity is thought to modulate neural functions, yet its impact on animal behavior remains elusive. Here, we reveal a potential role for prior activity in olfactory receptor neurons (ORNs) in shaping larval olfactory behavior. We show that prior activity in larval ORNs is mediated by the olfactory receptor complex (OR complex). Mutations of *Orco*, an odorant co-receptor required for OR complex function, cause reduced attractive behavior in response to optogenetic activation of ORNs. Calcium imaging reveals that *Orco* mutant ORNs fully respond to optogenetic stimulation but exhibit altered temporal patterns of neural responses. These findings together suggest a critical role for prior activity in information processing upon ORN activation in *Drosophila* larvae, which in turn contributes to olfactory behavior control.

## Introduction

Animals rely on their sensory neurons to detect external stimuli and change their behavior in response to the external world. However, sensory neurons are often activated prior to experiencing external stimuli. In many sensory systems, such neural activity prior to the stimulus, or prior activity, affects neural response to subsequent stimuli. For example, in the olfactory system of locusts, prior activity modulates response to odorants^1^. In addition, even in the absence of such background stimuli, many types of neurons exhibit spontaneous activity. For example, mitral cells in the mammalian olfactory bulb show patterned spontaneous activity in the absence of odor stimuli^2,3^. This spontaneous activity could also be regarded as a type of prior activity. These neural activities prior to stimuli are observed in many sensory systems and cortical regions^4–7^, yet their impacts on animal behavior remain unclear.

Olfactory receptor neurons (ORNs) of *Drosophila melanogaster* larvae provide an attractive model for studying functional links between neural activity and behavioral output. *Drosophila* larvae have only 21 ORNs that innervate the dorsal organs on each side of the head and project to individual glomeruli in the antennal lobe. Each ORN expresses its own olfactory receptor complex (OR complex) that consists of a cell-specific olfactory receptor (OR) and the co-receptor Orco^8^, with the cell-specific OR determining the odor specificity^9–11^. Activation of single ORNs induces either attractive or aversive behavior, indicating that the neural activity of each ORN tightly relates to stereotyped behavior. For example, larvae that have only a single pair of functional ORNs expressing Or42a exhibit robust attractive behavior to many odors^12^. By contrast, optogenetic stimulation of individual ORNs expressing Or33b, or Or45a, which detect aversive odors, induced aversive behavior toward the stimulating light^13^.

Within ORNs, Orco is a key regulator of both evoked and spontaneous activity. In ORNs, Orco regulates dendritic localization of ORs^8^ and forms heteromeric ligand-gated ion channels together with ORs^14,15^. Orco is therefore necessary for odor-evoked activity. In addition, Orco promotes spontaneous activity in ORNs. *Orco* mutation or pharmacological inhibition severely diminishes ORN spontaneous activity in *Drosophila*^8,16,17^ as well as a broad range of other insects, including *Anopholes gambiae*, and *Manduca sexta*^18,19^. While Orco expression may extend to tissues involved in other sensory modalities in some insects^20,21^, *Drosophila* Orco expression is apparently restricted to OR-expressing ORNs, thus *Orco* mutation provides a tool to potently and specifically block evoked and spontaneous activity in ORNs.

In this study, we aim to investigate potential roles for prior activity in the olfactory system by using *Drosophila* larval ORNs as the model system. We show that each larval ORN has different patterns of prior activity and that the OR complexes are the key components required for prior activity in larval ORNs. Optogenetic behavioral analysis indicates that *Orco* mutant larvae show reduced responses to ORN stimulation. Using a combination of optogenetic stimulation and *ex-vivo* calcium imaging, we show that *Orco* mutant ORNs exhibit altered temporal patterns of ORN activity in response to ORN stimulation. Our findings thus suggest a role for prior neural activity of ORNs in sensory processing and olfactory behavior control.

## Results

### Calcium imaging of larval olfactory receptor neurons

**Figure 1:**
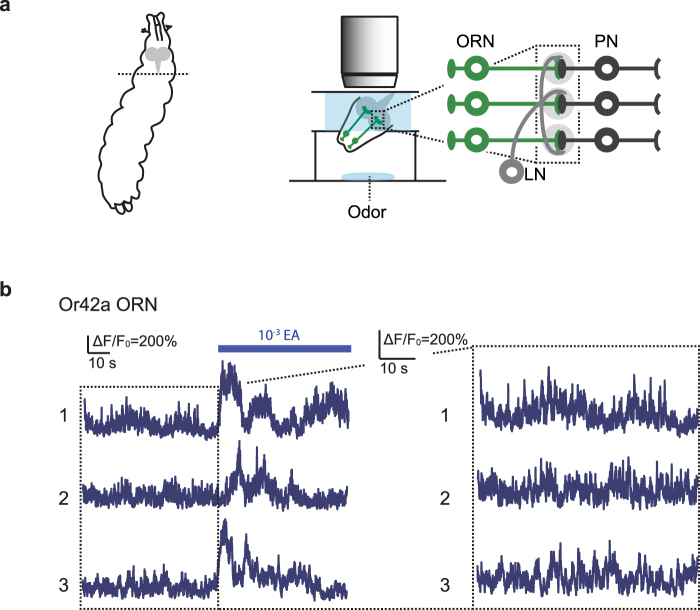
Calcium imaging of larval olfactory receptor neurons. (**a**) Schematic of the *ex vivo* calcium imaging preparation and the larval olfactory circuit. ORN, olfactory receptor neuron; LN, local neuron; PN, projection neuron. (**b**) Representative fluorescence changes from baseline (∆F/F_0_) of Or42a ORN in three different animals. 10^−3^ EA, 10^−3^ concentration of ethyl acetate. Genotype: *w*^1118^*;Or42a-Gal4*,*UAS-GCaMP6f/*+.

To examine neural activity in larval ORNs, we performed calcium (Ca^2+^) imaging of ORNs. To that end, we expressed the Ca^2+^ indicator GCaMP6f^22^ in ORNs using the Gal4-UAS system^23^ and monitored Ca^2+^ response in ORN axonal terminals in the larval brain (Fig. 1a). Consistent with a previous report^24^, we detected robust Ca^2+^ responses when the larval head was exposed to ethyl acetate, a strong attractant to larvae^9^ (Fig. 1b). In addition to the odor-evoked Ca^2+^ responses, we also observed continuous Ca^2+^ responses prior to the odor stimulation in ORNs (Fig. 1b). These responses may reflect spontaneous activity or responses to other background odorants; we subsequently refer to these responses as prior activity to reflect their presence prior to application of a defined odor. The prior activity in ORNs exhibited smaller amplitudes compared to the odor-evoked activity (Fig. 1b: Or42a, 61.1% of evoked activity on average, n = 11). In addition, video recordings of Ca^2+^ responses in ORNs demonstrated that the frequency of prior activity in each ORN is different from one another (Supplementary Movie S1). These data suggest that larval ORNs likely have different profiles of prior activity with unique frequency.

### Orco is essential for prior activity in larval ORNs

Given that firing patterns of prior activity seem to be different in each ORN, we reasoned that ORN-specific molecules might contribute to generating the prior activity. One potential candidate is the OR complexes as each of 21 ORNs in the larval olfactory organ (dorsal organ) expresses a unique set of OR complexes^25^. OR complexes are heteromeric ion channels that consist of the ORN-specific OR that determines the response spectrum of the ORN and the co-receptor Orco that is shared by most ORNs^14,15,26^.

**Figure 2:**
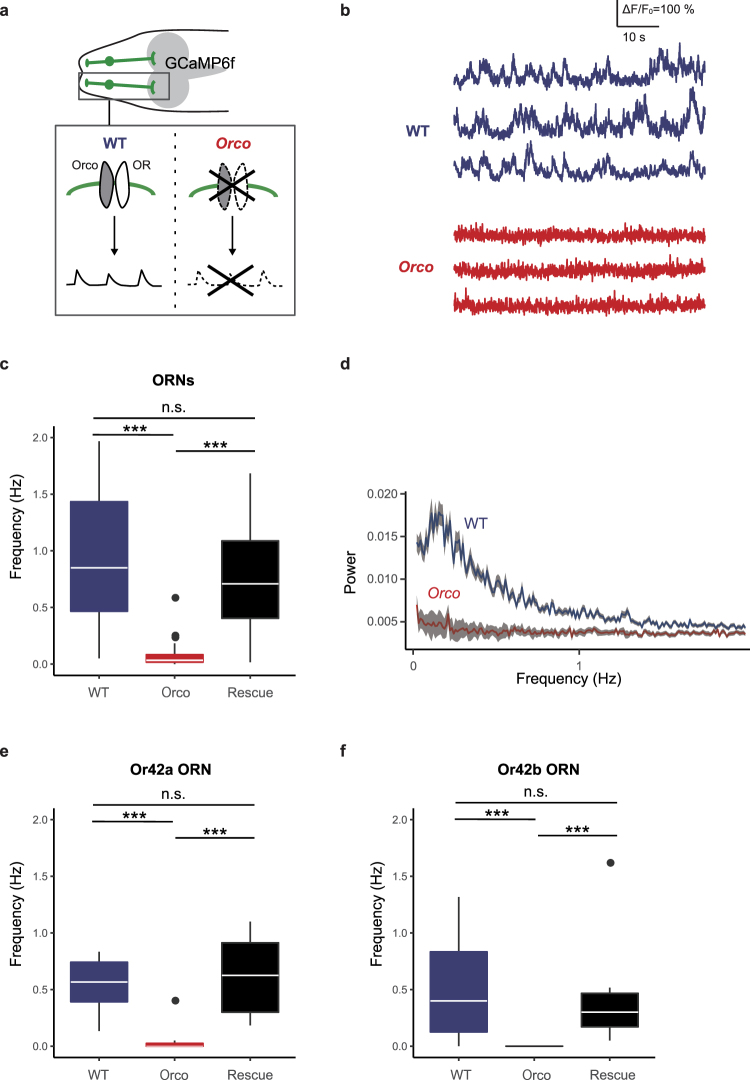
Orco is essential for prior activity in larval ORNs. (**a**) Schematic of Orco-OR channel of ORNs in wild-type (WT) and *Orco* mutant larvae. The channel contributes to the prior activity of ORNs. (**b**) Representative fluorescence changes from baseline (∆F/F_0_) of ORNs in wild-type (WT) and *Orco* mutant larvae. (**c**) Quantification of the frequency of the prior activity of ORNs in the wild-type (WT, blue; n = 57), *Orco* mutant (Orco, red; n = 57) and *Orco* expression-rescued strain (Rescue, black; n = 43). Box plots show the median (white line), 1st, and 3rd quartile (box), the data range (whiskers), and outliers (circles). Outliers are data points located outside the whisker range. (**d**) Average power spectra of the prior activity of ORNs in wild-type (WT, blue; n = 132) and *Orco* mutant (Orco, red; n = 118). Means ± SEM. Genotypes: WT, *w*^1118^*;Orco-Gal4*,*UAS-GCaMP6f/*+ Orco, *w*^1118^*;Orco-Gal4*,*UAS-GCaMP6f/*+*;orco*^1^*/orco*^1^. Rescue, *w*^1118^*;Orco-Gal4*,*UAS-GCaMP6f/UAS-Orco;orco*^1^*/orco*^1^. (**e** and **f**) Quantification of frequency of the prior activity of a single ORN, Or42a (**e**) and Or42b ORN (**f**). Genotypes: WT, *w*^1118^*;Or42a/b-Gal4/ UAS-GCaMP6f*. Orco, *w*^1118^*;Or42a/b-Gal4/UAS-GCaMP6f;orco*^1^*/orco*^1^. Rescue, *w*^1118^*;Or42a/b-Gal4*,*UAS-Orco/UAS-GCaMP6f;orco*^1^*/orco*^1^. ^***^P < 0.001, Wilcoxon rank-sum test with Bonferroni correction; n.s., not significant.

To examine potential roles for OR complexes in generation of ORN prior activity, we performed Ca^2+^ imaging in *Orco* mutant ORNs. *Orco* null mutations prevent OR complexes from localizing to ORN dendrites^8^ and Orco is required for formation of heteromeric channels with ORs. Hence, *Orco* mutation blocks odor-evoked responses of ORNs. *Orco* mutation additionally blocks spontaneous activity in embryonic and adult ORNs^16,17^. We likewise found that in *Orco* mutant ORNs prior activity was rarely detectable (Fig. 2a,b). Quantitative analysis indicated that the frequency of the prior activity in *Orco* mutant ORNs was significantly reduced compared to wild-type controls (Fig. 2c: WT, median 0.90, 1st quartile 0.48, 3rd 1.47; *Orco* mutants, median 0.060, 1st quartile 0.017, 3rd 0.083). We next performed power spectrum analysis of the prior activity and found that the characteristic peak observed in wild-type ORNs was largely absent in *Orco* mutant ORNs (Fig. 2d), indicating that not only the amplitude but also the rhythmicity of the prior activity was extinguished in *Orco* mutant ORNs. Recording the prior activity from single ORNs, Or42a and Or42b ORN, further revealed that the reduced prior activity in *Orco* mutant ORNs was detectable at the single neuron level (Fig. 2e,f). The reduced prior activity in *Orco* mutant ORNs was restored to the wild-type level by expressing *UAS*-*Orco* in ORNs (Fig. 2c,e and f). These data suggest that OR-complex expression in each ORN is essential for producing their unique prior activity as well as evoked activity in ORNs. While it is possible that Orco could directly influence membrane potential on its own, given that Orco family proteins can form functional cation channels in the absence of OR expression^14,15,19^, whether such channels are actually formed in ORNs remains to be determined.

### *Orco* mutant larvae exhibit reduced response to ORN activation

**Figure 3:**
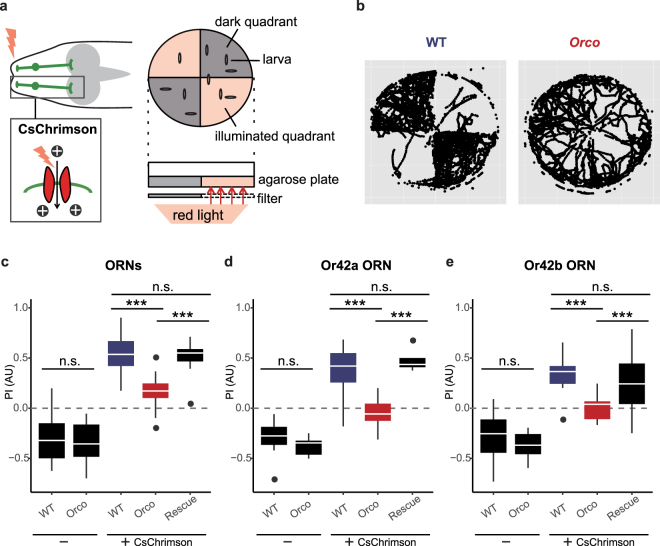
*Orco* mutant larvae exhibit reduced response to ORN activation. (**a**) Right, expression of CsChrimson in ORNs. Left, agarose gel arena for optogenetic choice assay. (**b**) Representative plots of WT (left) and *Orco* mutant (right) larval positions over a period of 5 min. (**c**) Performance index (PI) of larvae expressing CsChrimson in their ORNs. Genotypes: WT, *w*^1118^*;Orco-Gal4/UAS-CsChrimson*,*Tsh-Gal80*. Orco, *w*^1118^*;Orco-Gal4/UAS-CsChrimson*,*Tsh-Gal80;orco*^1^*/orco*^1^. Rescue, *w*^1118^*;Orco-Gal4*,*UAS-Orco/UAS-CsChrimson*,*Tsh-Gal80;orco*^1^*/orco*^1^. (**d** and **e**) Performance index (PI) of larvae expressing CsChrimson in a single ORN, Or42a (**d**) and Or42b (**e**). Genotypes: WT, *w*^1118^*;Or42a/b-Gal4/UAS-CsChrimson*,*Tsh-Gal80*. Orco, *w*^1118^*;Or42a/b-Gal4/UAS-CsChrimson*,*Tsh-Gal80;orco*^1^*/orco*^1^. Rescue, *w*^1118^*;Or42a/b-Gal4*,*UAS-Orco/UAS-CsChrimson*,*Tsh-Gal80;orco*^1^*/orco*^1^. ^***^P < 0.001, Wilcoxon’s rank-sum test with Bonferroni correction; n.s. not significant.

In order to investigate potential roles for the prior activity in larval behavior, we next examined whether *Orco* mutant larvae might have defects in olfactory behavior evoked by ORN activation. To activate wild-type and *Orco* mutant ORNs, we expressed the red light-gated cation channel CsChrimson^27^ in ORNs under the control of the *Orco*-*Gal4* driver and illuminated larvae with red light (617 nm) (Fig. 3a). We placed these transgenic larvae in the center of a Petri dish divided into quadrants receiving either red light illumination or no illumination (Fig. 3a). In this choice assay system, quadrants receiving red light illumination are bounded by dark quadrants, therefore ORNs expressing CsChrimson are optogenetically activated as long as larvae stay in the illuminated areas.

We first checked CsChrimson expression and found no significant difference in CsChrimson expression levels in wild-type and *Orco* mutant ORNs (Supplementary Fig. S1). We then performed the choice assay with wild-type larvae harboring CsChrimson in ORNs. Consistent with the previous reports^13^, wild-type larvae carrying CsChrimson in ORNs showed a strong preference to the illuminated areas compared to control larvae that tended to stay in the dark areas (Fig. 3b,c), suggesting that optogenetic activation of ORNs causes an attractive bias in larvae. We next examined *Orco* mutant larvae carrying CsChrimson in ORNs and found that, unlike wild-type larvae, *Orco* mutant larvae exhibited no significant preference between illuminated and dark areas (Fig. 3b,c). Similar data were obtained with wild-type and *Orco* mutant larvae expressing CsChrimson in single ORNs such as Or42a and Or42b, both of which are dominant ORNs activated by attractive odorants such as ethyl acetate^9,25^ (Fig. 3d,e). The reduced attraction to the illuminated areas in *Orco* mutant larvae was fully rescued to wild-type levels by expressing *UAS*-*Orco* in ORNs (Fig. 3c–e), suggesting the cell-autonomous role of OR complexes in ORNs. Together with our finding that OR complexes are essential components to generate the prior activity in ORNs, these observations suggest potential roles for the prior activity of ORNs in control of olfactory behavior evoked by ORN activation.

### *Orco* mutant larvae are defective in turning behavior at the light/dark boundary

**Figure 4:**
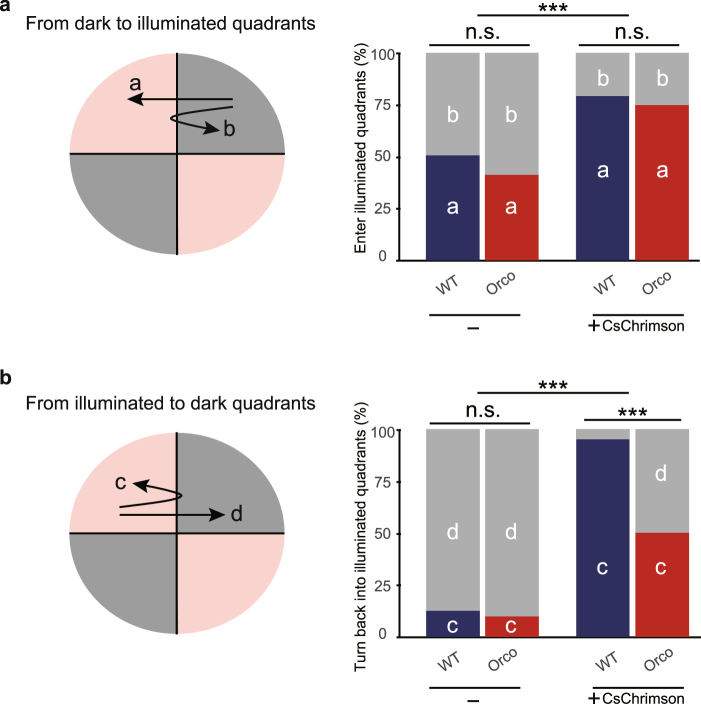
*Orco* mutant larvae are defective in turning behavior at the light/dark boundary. (**a**) Probability of entering illuminated quadrants from dark quadrants. (**b**) Probability of turning back into illuminated quadrants. Genotypes: WT, *w*^1118^*;Orco-Gal4/UAS-CsChrimson*, *Tsh-Gal80*. Orco, *w*^1118^*;Orco-Gal4/UAS-CsChrimson*,*Tsh-Gal80;orco*^1^*/orco*^1^. ^***^P < 0.001, Fisher’s exact test with Benjamini−Hochberg correction^46^; n.s. not significant.

What behavioral changes might cause *Orco* mutant larvae to spend less time in illuminated areas compared to wild-type? To address this, we analyzed larval behavior at the boundary of illuminated and dark quadrants. First, we examined behavior of larvae moving from the dark to the illuminated areas. Video recording of individual traces indicated that ~75% of larvae harboring CsChrimson passed into the illuminated areas in both wild-type and *Orco* mutant larvae, while ~50% of wild-type and *Orco* mutant larvae lacking CsChrimson crossed the boundaries (Fig. 4a). These data indicate that loss of OR-complex functions has no significant effect on larval moving from the dark to the illuminated areas.

We next focused on the larvae moving from the illuminated quadrants toward the dark quadrants. Almost 100% of wild-type larvae harboring CsChrimson in ORNs made an obvious turn when they approached from the illuminated to the dark areas (Fig. 4b; 95.5%, n = 49). In contrast, only ~50% *Orco* mutant larvae harboring CsChrimson passed into the dark areas (Fig. 4b; 51.0%, n = 69). These observations suggest that the reduced preference to the illuminated areas in *Orco* mutant larvae is attributed at least in part to the reduced turning behavior at the boundary when they move from the illuminated quadrants to the dark quadrants.

### Temporal dynamics of ORN response to continuous stimulations are altered in *Orco* mutant larvae

**Figure 5:**
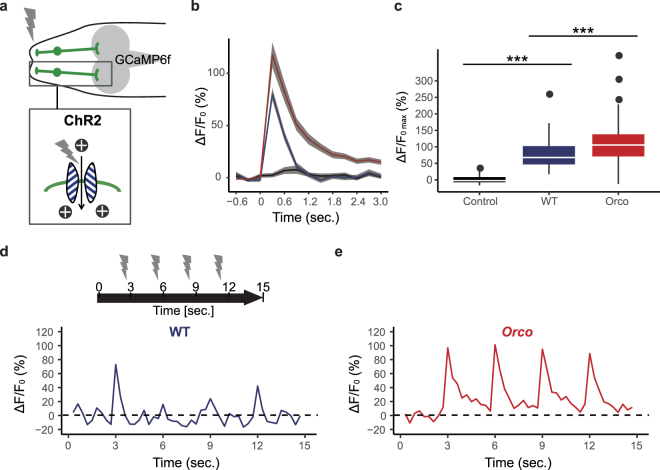
Temporal dynamics of ORN response to pulse stimulations. (**a**) Expression of ChR2 and GCaMP6f in ORNs. (**b**) Average fluorescence changes (∆F/F_0_) to blue light stimulation in WT (blue; n = 64), Orco mutant (red; n = 40), and control larvae that did not carry *UAS-ChR2* (black; n = 50). Means ± SEM. (**c**) Quantification of maximum ∆F/F_0_ (∆F/F_0max_) of ORNs in the control, WT, and *Orco* mutant larvae. (**d** and **e**) Representative traces of ∆F/F_0_ of a single ORN in WT (**d**) and *Orco* mutant (**e**). ORNs are stimulated with the blue light pulses at four time points. Genotypes: Control, *w*^1118^*;Orco-Gal4*,*UAS-GCaMP6f/Orco-Gal4*. WT, *w*^1118^*;Orco-Gal4*,*UAS-GCaMP6f/Orco-Gal4*,*UAS-hChR2[H134R]*. Orco, *w*^1118^*;Orco-Gal4*,*UAS-GCaMP6f/Orco-Gal4*,*UAS-hChR2[H134R];orco*^1^*/orco*^*1*^.

To address how the prior activity in ORNs could affect the turning behavior, we examined neural responses of ORNs evoked by optogenetic stimulation in wild-type and *Orco* mutant larvae. We first tested whether *Orco* mutant ORNs could respond to single optogenetic stimulations. We simultaneously expressed channelrhodopsin-2 (ChR2)^28^ and GCaMP6f in ORNs and measured Ca^2+^ responses induced by blue light stimulation of larvae (Fig. 5a). A previous report also showed that larvae expressing ChR2 in their ORNs were attracted to the blue-light stimulation^29^. Thus, behavioral and neural responses to ChR2 likely mirror the responses to CsChrimson. Blue light application induced Ca^2+^ responses in both wild-type and *Orco* mutant ORNs (Fig. 5a). Intriguingly, the average amplitude of Ca^2+^ responses was higher in *Orco* mutant ORNs than in wild-type (Fig. 5b,c: wild-type, median 67.6%, 1st quartile 46.7%, 3rd quartile 67.6%, n = 64; *Orco* mutants, median 105.6%, 1st quartile 71.1%, 3rd quartile 138.0%, n = 34). Furthermore, *Orco* mutant ORNs consistently responded to repeated blue light applications, whereas wild-type ORNs exhibited large variability in the amplitude of their responses (Fig. 5d,e). These data indicate that *Orco* mutant ORNs respond to stimulation better than wild-type, and it is thus unlikely that reduced preference toward illuminated areas in *Orco* mutant larvae is simply due to reduced excitability in *Orco* mutant ORNs.

**Figure 6:**
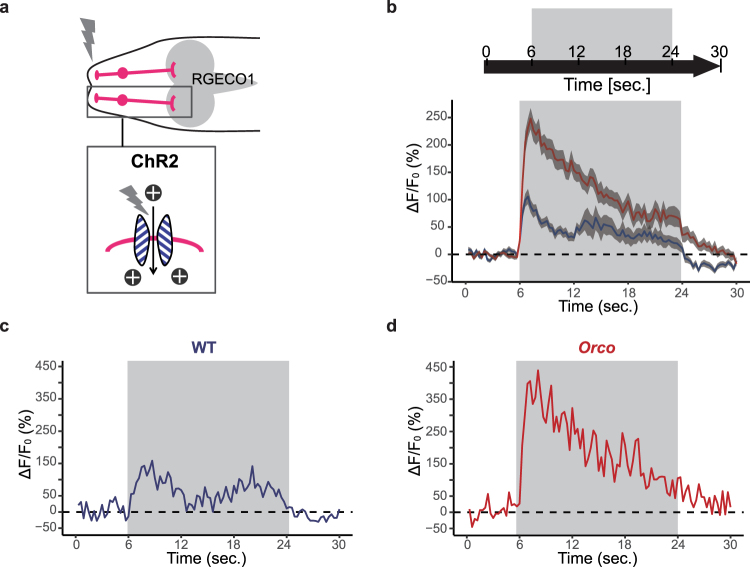
Temporal dynamics of ORN response to continuous stimulations are altered in *Orco* mutant larvae. (**a**) Expression of ChR2 and RGECO1.0 in ORNs. (**b**) Average ∆F/F_0_ to blue light stimulation in WT (blue; n = 31) and *Orco* mutants (red; n = 33). Means ± SEM. (**c** and **d**) Representative traces of ∆F/F_0_ of a single ORN in WT (**c**) and *Orco* mutant (**d**). ORNs are stimulated with the blue light for 18 s. Genotypes: WT, *w*^1118^*;Orco-Gal4*,*UAS-RGECO*1*/Orco-Gal4*,*UAS-hChR2 [H*1*34R]*. Orco, *Orco-Gal4*,*UAS-RGECO1/Orco-Gal4*,*UAS-hChR2[H134R];orco*^1^*/orco*^1^.

Next, we examined how *Orco* mutant ORNs could respond to continuous stimulation. This stimulation paradigm likely reflects our behavioral assay rather than the pulse stimulations, as *Orco* mutant larvae exhibited turning defects when they encountered the light-dark boundary after continuous ORN stimulations in the illuminated areas (Fig. 4). Similar to the pulse stimulations (Fig. 5), *Orco* mutant ORNs responded to continuous stimulations more strongly than wild-type ORNs at the onset of the stimulations (Fig. 6a–d). However, during the course of the continuous stimulation, Ca^2+^ responses in *Orco* mutant ORNs gradually decreased, whereas Ca^2+^ responses in wild-type ORNs exhibited some fluctuation but were sustained at a certain level (Fig. 6b–d). Furthermore, wild-type and *Orco* mutant ORNs exhibited distinct responses to termination of prolonged stimulation. In wild-type ORNs, Ca^2+^ responses were quickly diminished to levels below the pre-stimulus baseline, whereas Ca^2+^ responses in *Orco* mutant ORNs were gradually reduced but did not return to the baseline for an extended period of time (Fig. 6b–d). Given that reduction of the turning behavior in *Orco* mutant larvae was specifically observed at the light-dark boundary where the larvae suddenly lost continuous ORN stimulation, the different neural responses right after termination of the continuous stimulation in wild-type and *Orco* mutant ORNs might contribute to the changed turning behavior upon ORN activation.

One might imagine that ion channel gene expression in *Orco* mutant ORNs could account for the different patterns of activation. To test this, we assayed for changes in ion channel gene expression between wild-type and *Orco* mutant dorsal organs using RNA-Seq analysis (Supplementary Fig. S2a), and found no significant changes in ion channel gene expression in *Orco* mutant dorsal organs; among 148 ion channel genes queried, only *Orco* and 4 *OR* genes exhibited significant differential expression between wild-type and Orco mutant samples (Supplementary Fig. S2b, Supplementary Table 1). These results suggest that the differences in neural responses in *Orco* mutant ORNs are unlikely to be caused by changes in ion channel expression.

### PNs respond to ORN stimulations in *Orco* mutant larvae

In the *Drosophila* olfactory system, projection neurons (PNs) relay olfactory information from ORNs to higher-order brain areas^30,31^. To confirm that the neural responses of ORNs are transferred to PNs in *Orco* mutants, we explored whether functional synapses were formed between ORNs and PNs. To this end, we expressed the presynaptic marker, Bruchpilot::GFP (Brp::GFP)^32^, in Or42a ORNs and observed the localization of the marker signals. We found that the Brp signals were localized at the axonal terminals in both wild-type and *Orco* mutant ORNs, though the signals in *Orco* mutant ORNs seemed to be more condensed compared to wild-type (Supplementary Fig. S3). This result suggests that the synapses between ORNs and PNs are formed in *Orco* mutants.

**Figure 7:**
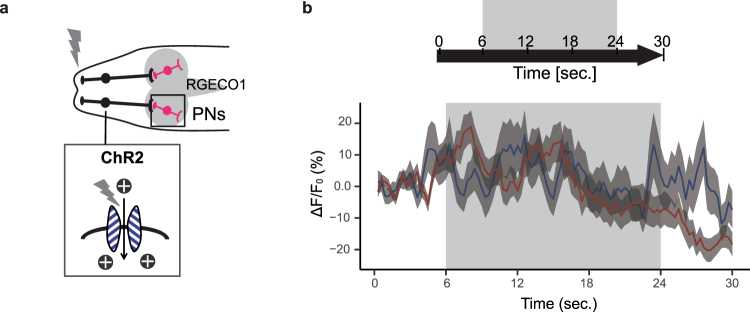
PNs respond to continuous stimulations of ORNs in *Orco* mutant larvae. (**a**) Expression of ChR2 in ORNs and RGECO1.0 in PNs. (**b**) Average ∆F/F_0_ of PNs to blue light stimulation of ORNs in wild-type (blue; n = 56) and *Orco* mutants (red; n = 70). Means ± SEM. Genotypes: WT, *w*^1118^*;GH*1*46-Gal4*,*Orco-ChR2/UAS-RGECO*1,*Orco-ChR2*. Orco, w^1118^;*GH146-Gal4*,*Orco-ChR2/UAS-RGECO1*,*Orco-ChR2;orco*^1^*/orco*^1^.

Next, we examined whether PNs could respond to continuous stimulations of ORNs. We expressed ChR2 in ORNs and the Ca^2+^-responsive red fluorescent protein RGECO1 in PNs, and measured PN Ca^2+^ responses induced by blue light stimulation of ORNs (Fig. 7). The PN responses were similar to ORN responses in regard to temporal patterns during stimulation. At the onset of the ORN activation, the response of PNs was larger in *Orco* mutant compared to wild-type. However, PN responses in *Orco* mutants were gradually decreased and finally became weaker compared to wild-type. These results suggest that functional connectivity between ORNs and PNs is likely retained in *Orco* mutants.

## Discussion

To realize proper sensory processing, neural circuits in the brain decode evoked responses based on the neural activity prior to the stimulation. Such prior activity is generally observed in olfactory, auditory, visual, and cortical neurons^5,7,33–35^. In certain cases, the prior activity is thought to modify the evoked response to stimuli^34–37^. However, the contribution of prior activity to output behavior remains largely unknown because of difficulty in manipulating prior activity *in vivo* and lack of a simple model for assessing the effect of prior activity.

In this study, we have provided the following evidence that support the potential role for prior activity in control of olfactory behavior. First, we showed that prior activity in ORNs is likely mediated at least in part by OR complexes, as prior activity was significantly reduced in *Orco* mutant ORNs (Fig. 2). Second, we found that *Orco* mutant larvae, which had reduced prior activity in ORNs, showed less attractive behavior to illuminated areas in a choice test arena in which illumination drives optogenetic activation of ORNs (Fig. 3). Third, we demonstrated that the behavioral changes in *Orco* mutant larvae were attributed to reduced turning behavior when larvae approached the boundary from the light area (Fig. 4). The behavioral defects in *Orco* mutant larvae were fully rescued by introducing one copy of *Orco* gene in ORNs, indicating the cell-autonomous function of Orco in ORNs (Fig. 3).

How could the prior activity mediated by OR complexes affect the turning behavior? It seems unlikely that the prior activity affects ORN excitability as *Orco* mutant ORNs showed substantial activity in response to optogenetic activation (Fig. 5). One possibility is that the prior activity could directly modify the firing patterns of ORNs upon activation. Indeed, temporal patterns of the optogenetically-evoked activity in wild-type larvae seemed to fluctuate probably due to the prior activity, whereas *Orco* mutant ORNs exhibited consistent firing patterns upon activation (Fig. 6).

An alternative but not mutually exclusive scenario is that the prior activity might affect expression levels of functional proteins, such as ion channels. Temporal patterns of neuronal activity are basically determined by the combinatory activities of multiple ion channels^38^. While our RNA-Seq analysis showed that the expression levels of ion channels were not significantly different between wild-type and *Orco* mutant ORNs, *Orco* mutant ORNs exhibited different temporal patterns of neural responses from wild-type ORNs (Fig. 6). Thus, one possibility is that prior activity might regulate post-transcriptional control of channel expression or modulate ion channel function by another mechanism, such as post-translational modification and/or trafficking, and hence change the temporal pattern of neural responses. Another possibility is that connectivity between ORNs and PNs might be influenced by the prior activity. Previous studies suggested that prior activities in neurons, including spontaneous activity, affect synaptic formation^39–41^. Furthermore, blocking neural activity of ORNs led to alterations in the morphology of ORN axon terminals in *Drosophila* embryos^16^. Consistent with these results, we observed alterations in the distribution of presynaptic marker signals in axonal terminals of *Orco* mutant ORNs compared to wild-type ORNs (Supplementary Fig. S3). We, therefore, cannot exclude the possibility that prior activity might modify the connectivity between ORNs and PNs. However, *Orco* mutant PNs responded to continuous ORN activation, suggesting that functional connectivity between ORNs and PNs in *Orco* mutants is intact. Thus, deprivation of prior activity in *Orco* mutant ORNs might affect neural responses in *Orco* mutant at the circuit level, which in turn modulate the animal behavior upon ORN activation.

It is also feasible that the prior activity might play a role in inactivation of ORNs, as *Orco* mutant larvae failed to turn at the boundary specifically when larvae moved from illuminated areas in which ORNs were optogenetically activated to dark areas (Fig. 4). Ca^2+^ imaging data indicate that optogenetically-evoked activity in wild-type ORNs was quickly suppressed to the basal level once larvae passed the boundary from illuminated areas to the dark areas, whereas *Orco* mutant ORNs required longer times for evoked activity to return to the basal level (Fig. 6). Considering the results of the behavioral assay, these observations indicate that the turning behavior at the boundary may be induced by the transition of the response amplitude from a certain level to below the baseline. Consistent with this possibility, larvae expressing CsChrimson in Or42a ORNs demonstrated increased run-to-turn transitions when optogenetic activation was reduced^42^. Based on this notion, prior activity might, at least in part, contribute to the reduction of neural response of ORNs. Thus, the prior activity might be one of the critical factors for suppression of neural responses.

In summary, by the use of a combined optogenetic and *ex vivo* imaging technique in *Drosophila* larval ORNs, we have revealed a potential role for prior activity in control of olfactory behavior evoked by ORN activation. Given its relative simplicity, combined with the powerful genetic tools in *Drosophila*, our system could contribute to elucidation of the molecular and circuit-based mechanisms of how prior activity in ORNs regulates animal behavior.

## Materials and Methods

### Drosophila strains

Fly strains used in the present study were as follows: *Orco*-*Gal4* (Bloomington #26818), *UAS-GCaMP6f* (Bloomington #42747), *w*^*−*^*;; w*^+^, *orco*^1^ (null allele, Bloomington #23129), *Or42a-Gal4* (Bloomington #9970), *Or42b-Gal4* (Bloomington #9972), *UAS-Orco* (second, this study), *UAS-CsChrimson* (Bloomington #55135), *Tsh-Gal80* (second, kindly gifted by Dr. Miesenböck), *UAS-RGECO1* (our stock)^43^, *UAS-hChR2[H134R]* (our stock)^43^, *Orco-ChR2* (Bloomington #63041)^44^, *GH146-Gal4* (Bloomington #30026).

### Transgenic strain

The *Orco* cDNA was PCR amplified from a plasmid (kindly provided by Dr. Vosshall) and then subcloned into the pJFRC-MUH-20xUAS vector^43^. The plasmid was injected into flies carrying the attP40 docking site (BestGene Inc.).

### Calcium imaging

*Ex vivo* calcium imaging was performed as previously described^24^. Larvae were raised with the standard fly food at 25°C under 12-h light/dark conditions. Third instar larvae were washed in 1 × PBS and transferred to adult hemolymph-like (AHL) saline^45^. To expose the larval brain, each larva was dissected, and the larval head was isolated by removing the fat body, the salivary gland, and the digestive system. The nose tip of the larval head was inserted into the hole of 22 mm $\times$ 22 mm plastic cover slip (Thermo Fisher Scientific) that was punched with 23 G needle (Terumo Corporation). To fix the brain position, the brain was covered with low-melting agarose (1.0%; LowMelt Agarose, GeneMate) in AHL and coverglass. For odor stimulation, 10 µl ethyl acetate at 10^−3^ dilution was applied manually.

Calcium imaging was performed using an Olympus BX51WI (UPlanSApo objective, 40×, NA = 0.75, Olympus), with a Yokogawa CSU10 (Yokogawa, Tokyo, Japan) spinning disk confocal system and an electron-multiplying charge-coupled device (EM-CCD) digital camera (Evolve, Photometrics)^43^. The GCaMP6f signal was obtained at about 20 frames s^−1^ (exposure time, 50 msec). Motion correction of the obtained movies was performed using Metamorph (Molecular Devices). For calculation of the signal intensities, a region of interest (ROI) was manually selected using Image J software (http://rsb.info.nih.gov/ij/). F_0_ was computed as the mean of the 25th percentile of signal intensities in each ROI. ∆F/F_0_ was calculated according to (F-F_0_)/F_0_. For peak detection, σ was calculated from a ΔF/F_0_ time series. Peaks were then detected as events that deviated 2σ from the baseline. Peaks were detected and the power spectrum was calculated using MATLAB (http://www.mathworks.com/). For statistical analysis of the frequency of prior activities, Wilcoxon’s rank-sum test, followed by Bonferroni correction, was applied.

### Optogenetic choice assay

Larvae were raised with the standard fly food containing 1 mM all-trans-retinal at 25 °C in the dark. Third instar larvae were washed in water and transferred into 10-cm diameter agarose gel (Nippon gene) plates. Ten larvae were placed at the center of the plate. This plate was put in the dark experimental chamber. In this chamber, the agarose plate was illuminated by red light. Larvae expressing CsChrimson were allowed to move between two dark quadrants and two illuminated quadrants on the plate. For optogenetic stimulation, larvae on the plate were exposed to light (617 nm, ~35 *μ*W/mm^2^; M617L3, THORLABS). The larval behaviors were recorded at about 1 frame s^−1^ for 5 min by means of a CCD Camera (1500M-GE, THORLABS). The positions of larvae on the plate were analyzed using Image J software. The performance index (PI) was calculated as the number of larval positions detected in the illuminated area, minus the number of larval positions detected in the dark area, divided by the total number of larvae, over a period of 5 min. For statistical analysis of PIs, Wilcoxon’s rank-sum test, followed by Bonferroni correction, was applied. Entering and turning behaviors at the boundary were counted manually. The probability of entering illuminated quadrants from dark quadrants was calculated as the number of larvae that entered illuminated quadrants, divided by the total number of larvae that crossed the boundary from dark quadrants into illuminated quadrants. The probability of turning back into illuminated quadrants was calculated as the number of larvae that turned back into the illuminated quadrants, divided by the total number of larvae that crossed the boundary from the illuminated quadrants into the dark quadrants. For statistical analysis of these probabilities, Fisher’s exact test, followed by Benjamini−Hochberg correction^46^, was applied.

### Optical stimulation

Larvae were raised with the standard fly food containing 1 mM all-trans-retinal at 25 °C in the dark. The dissection of larvae and the sample preparation were described in the calcium imaging section. The stimulation and recording were performed with an Olympus BX51WI upright microscope (UPlanSApo objective, 40×, NA = 0.75, Olympus). For stimulation of ORNs, blue light was illuminated by pE-100 (CoolLED). GCaMP6f and RGECO1 signals were obtained at about 5 frames s^−1^ (exposure time, 200 ms). Obtained images were essentially analyzed as described in the calcium imaging section. In addition, background correction was performed to reduce the background noise by light stimulation. The images were mean-filtered (radius: 2 pixels) and then the background was subtracted using the rolling ball method (rolling ball radius: 50 pixels). For calculation of the fluorescence intensities, a region of interest (ROI) was manually selected using Image J software (http://rsb.info.nih.gov/ij/). F_0_ was computed as the mean intensity of nine frames from stimulation onset in each ROI. ∆F/F_0_ was calculated as (F-F_0_)/F_0_.

## Electronic supplementary material

Supplemental Movie S1

Supplemental information

Supplemental dataset for RNAseq
