# Supplementary material for: Prior activity of olfactory receptor neurons is required for proper sensory processing and behavior in Drosophila larvae
Source: Sci Rep. 2018 Jun 5;8:8580. doi: 10.1038/s41598-018-26825-3 (PMC5988719; doi:10.1038/s41598-018-26825-3)
Supplement: Supplementary file 2 — Supplemental information [file 41598_2018_26825_MOESM2_ESM.pdf]

Supplementary information

Prior activity of olfactory receptor neurons is required for proper sensory processing and behavior in *Drosophila* larvae

Nao Utashiro[1], Claire R. Williams[2, 3], Jay Z. Parrish[2, 3], and Kazuo Emoto[1, 4, #]

[1]Department of Biological Sciences, School of Science, The University of Tokyo
7-3-1 Hongo, Bunkyo-ku, Tokyo113-0033 Japan

[2]Department of Biology, University of Washington
24 Kincaid Hall, Box 351800, Seattle, WA 98195 USA

[3]Molecular and Cellular Biology Program, University of Washington, Seattle, WA, 98195, USA.

[4]International Research Center for Neurointelligence (WPI-IRCN), The University of Tokyo, 7-3-1 Hongo, Bunkyo-ku, Tokyo113-0033 Japan

[#]Correspondence: emoto@bs.s.u-tokyo.ac.jp
TEL: +81-3- 5841-4426 FAX: +81-3- 5841-4632

Supplementary methods

Drosophila strains

Fly strains used in the supplementary figures were as follows: *UAS-mCD8::RFP* (second, Bloomington #27398), *UAS-mCD8::GFP* (second, Bloomington #5137), *UAS-Brp::GFP* (first, Bloomington #35848), $w^{-};;w^{+},orco^{2}$ (null allele, Bloomington #23130)

Confocal imaging

Larvae were raised with the standard fly food at 25 ˚C under dark conditions. For dorsal organ imaging, third instar larvae were washed and dissected in 1 × PBS, and dorsal organs were mounted in Vectashield mounting medium (Vector Laboratories). Images were obtained by confocal microscopy (Leica TCS SP8). For larval brain imaging, larval brains were fixed by 4% formaldehyde/PBS for 30 min at room temperature. After fixation, the brains were transferred in PBS containing 0.1% Triton X-100 and incubated at 4 ˚C for four hours. These samples were mounted in Vectashield mounting medium. Images were obtained by confocal microscopy (Leica TCS SP8).

Immunostaining

For normalization of CsChrimson::Venus signal, 3rd instar larvae were dissected and fixed in 4% hormaldehyde/PBS and then stained with nc82 antibody (1:50; Developmental Studies Hybridoma Bank). As secondary antibody, Goat anti-mouse IgG Alexa Fluor 635 (1:500; Life technologies) was used. The samples were mounted

in Vectashield mounting medium (Vector Laboratories).

RNA-Seq Library Preparations

Four control and six *Orco* mutant samples with 20 dorsal organs each were isolated and subjected to mRNA-Seq analysis. Thoracic segments containing chemosensory organs were dissected from third instar larvae expressing *UAS-mCD8-GFP* in ORNs under control of *Orco-Gal4*. Experimental genotypes were: w^{1118}*;Orco-Gal4,UAS-mCD8-GFP/Orco-Gal4,UAS-mCD8-GFP* (control); w^{1118}*;Orco-Gal4,UAS-mCD8-GFP/Orco-Gal4,UAS-mCD8-GFP;*$orco^{1}/orco^{1}$ (*Orco* mutant). Dissected tissue was enzymatically treated with type I collagenase (Fisher), GFP-positive dorsal organs were manually separated from neighboring tissue and snap frozen in RNAqueous lysis buffer (Fisher). RNA was isolated using the RNAqueous Micro Kit, diluted to 0.2ng/ul, and converted to pre-amplified cDNA libraries using Smart-seq2 template-switching reverse transcription[1]. cDNA libraries were fragmented, barcoded, and amplified using the Nextera XT DNA kit, and all libraries were pooled and purified using AMPure XP beads. Quality was confirmed on an Agilent Bioanalyzer and libraries were sequenced on a HiSeq 4000 at the UCSF Center for Advanced Technology as 51 base single-end reads.

RNA-Seq Data Analysis

Reads were demultiplexed with CASAVA (Illumina) and read quality was assessed with FastQC (http://www.bioinformatics.babraham.ac.uk/projects/fastqc/). One *orco*

sample was excluded from analysis due to low read count and the remaining samples had read counts ranging from five to eleven million reads. Reads containing adapters were removed using cutadapt (version 1.9.1)[2]. To generate high recall of differentially expressed genes[3], reads were aligned to the *D. melanogaster* transcriptome, FlyBase genome release 6.10, using Kallisto (version 0.43.0)[4] with default parameters. Transcript-level counts were condensed to gene-level counts using tximport[5] and differential expression analysis was performed using DESeq2 (version 1.14.1)[6]. A Benjamini & Hochberg multiple comparison adjustment[7] was run on the original p-values to specifically assess differential expression for ion channel genes (identified as members of the Flybase Ion channel gene group, FBgg0000582; Supplemental Table 1). Genes with an adjusted p-value of under 0.05 were considered significantly different. The raw sequencing reads and gene expression estimates are available in the NCBI Sequence Read Archive (SRA) and in the Gene Expression Omnibus (GEO), accession number pending.

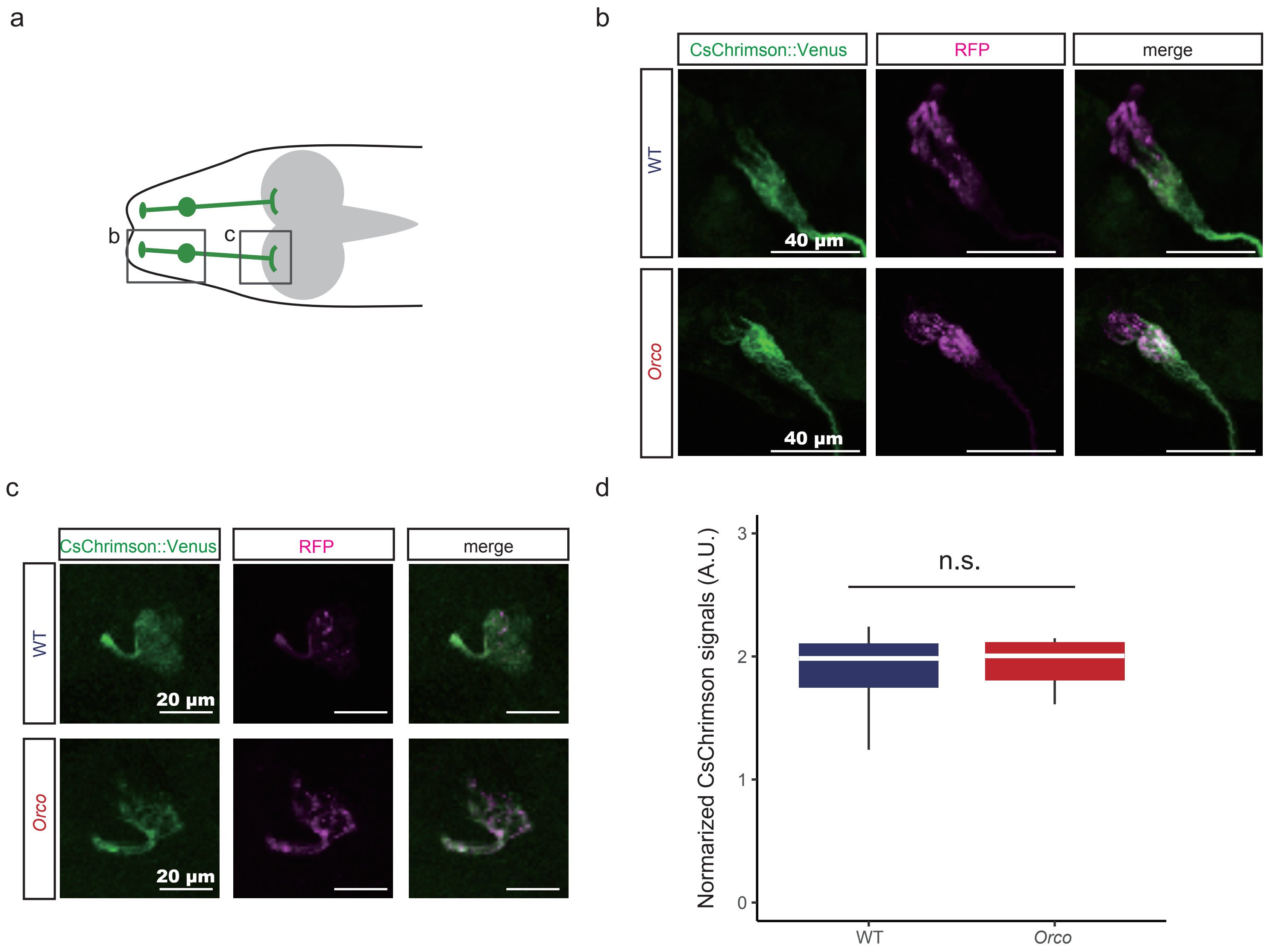

Supplementary Fig. S1

(a) Schematic shows ORNs in larval head. (b and c) *CsChrimson::Venus* (green) expression in dendrites (b) or axonal terminals (c) of ORNs. ORNs were visualized by *mCD8::RFP* (magenda). (d) Quantification of *CsChrimson::Venus* signals normalized to nc82 staining at the axonal terminals. Wilcoxon' s rank-sum test; n.s. not significant.

Genotype: WT, *w*^{1118}*;Orco-Gal4,UAS-CD8::RFP/UAS-CsChrimson::Venus,Tsh-Gal80*.

Orco, *w*^{1118}*;Orco-Gal4,UAS-CD8::RFP/UAS-CsChrimson,Tsh-Gal80;orco*^{1}*/orco*^{1}.

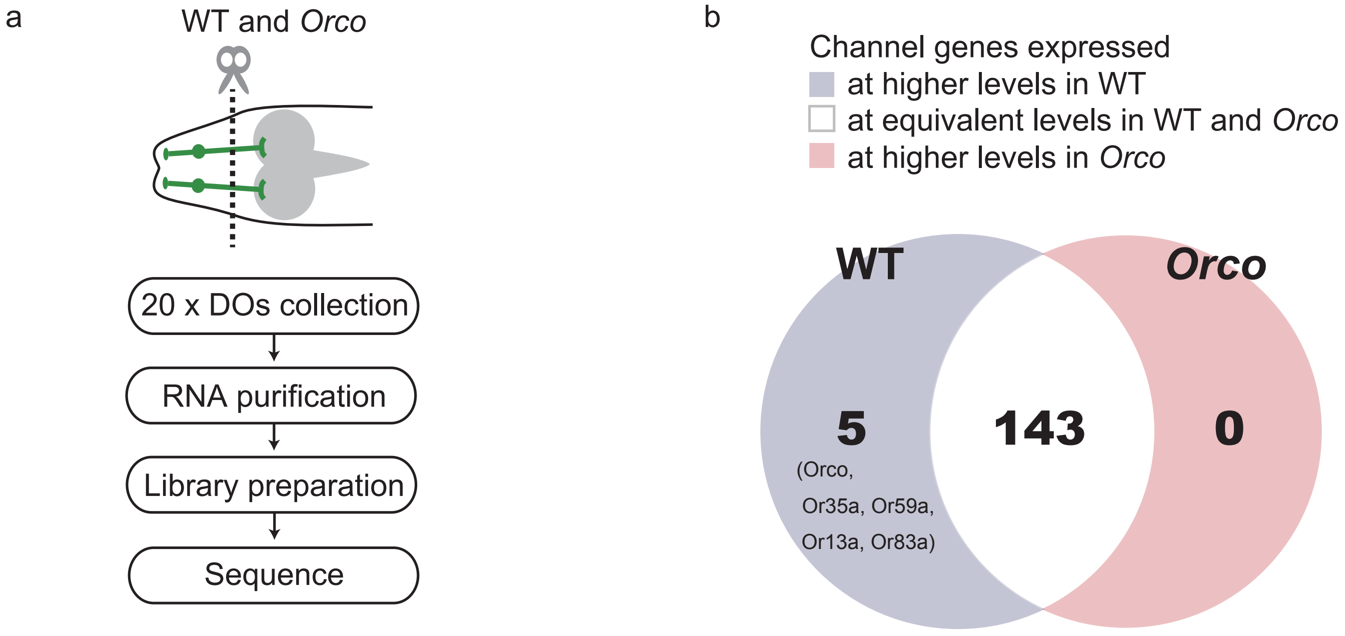

Supplementary Fig. S2

(a) Schematic Diagram of RNAseq analysis. DOs indicates dorsal organs. A detailed description of the library preparation protocol is provided in the supprementary methods. (b) Differential expression (DE) analysis of ion channel gene expression in wild-type and *Orco* mutant dorsal organs. Venn diagram shows the number of ion channel genes that were expressed at significantly higher levels in WT (Blue), at higher levels in *Orco* mutants (Red), or at equivalent levels in WT and *Orco* mutant dorsal organs (White). 127 genes were excluded from DE analysis due to insufficient information for expression estimation. The gene list and results of differential expression analysis are available in Supplementary Table S1, details on the methodology are available in the supplemental methods. Genotype: WT, *w^{1118};Orco-Gal4,UAS-CD8::GFP/Orco-Gal4,UAS-CD8::GFP*.

Orco, *w^{1118};Orco-Gal4,UAS-CD8::GFP/Orco-Gal4,UAS-CD8::GFP;$orco^{1}$/$orco^{1}$*.

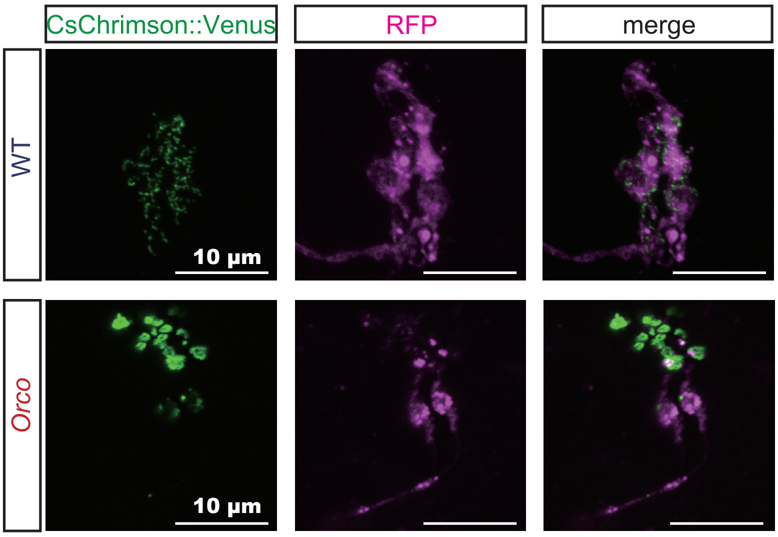

Supplementary Fig. S3

Brp::GFP (green) expression in axonal terminals of *Or42a* ORNs that additionally express *mCD8::RFP* (magenda). Genotype: WT, w^{1118}*;Or42a-Gal4,UAS-CD8::RFP/UAS-Brp::GFP*.

Orco, w^{1118}*;Or42a-Gal4,UAS-CD8::RFP/UAS-Brp::GFP;orco*^{1}*/orco*^{2}.

Supplementary references

1. Picelli, S. *et al.* Full-length RNA-seq from single cells using Smart-seq2. *Nat. Protoc.* **9,** 171–181 (2014).
2. Martin, M. Cutadapt removes adapter sequences from high-throughput sequencing reads. *EMBnet.journal* **17,** 10 (2011).
3. Williams, C. R., Baccarella, A., Parrish, J. Z. & Kim, C. C. Empirical assessment of analysis workflows for differential expression analysis of human samples using RNA-Seq. *BMC Bioinformatics* **18,** 38 (2017).
4. Bray, N. L., Pimentel, H., Melsted, P. & Pachter, L. Near-optimal probabilistic RNA-seq quantification. *Nat. Biotechnol.* **34,** 525–527 (2016).
5. Soneson, C., Love, M. I. & Robinson, M. D. Differential analyses for RNA-seq: transcript-level estimates improve gene-level inferences. *F1000Research* **4,** 1521 (2015).
6. Love, M. I., Huber, W. & Anders, S. Moderated estimation of fold change and dispersion for RNA-seq data with DESeq2. *Genome Biol.* **15,** 550 (2014).
7. Benjamini, Y. & Hochberg, Y. Controlling the False Discovery Rate: A Practical and Powerful Approach to Multiple Testing. *Journal of the Royal Statistical Society. Series B (Methodological)* **57,** 289–300 (1995).
